# Heated Intraperitoneal Chemotherapy in the Management of Advanced Ovarian Cancer

**DOI:** 10.3390/cancers10090296

**Published:** 2018-09-01

**Authors:** Andrea Jewell, Megan McMahon, Dineo Khabele

**Affiliations:** Department of Obstetrics and Gynecology, Division of Gynecologic Oncology, University of Kansas Medical Center, Kansas, KS 66160, USA; mmcmahon3@kumc.edu (M.M.); dkhabele@kumc.edu (D.K.)

**Keywords:** ovarian cancer, heated intraperitoneal chemotherapy (HIPEC), intraperitoneal chemotherapy (IP), cytoreductive surgery, secondary cytoreduction, interval cytoreduction

## Abstract

Heated intraperitoneal chemotherapy (HIPEC) has several potential benefits. Higher doses of chemotherapy can be used with HIPEC because the plasma-peritoneal barrier results in little absorption into the blood stream. HIPEC offers higher peritoneal penetration in comparison to an intravenous (IV) regimen and does not have the traditional normothermic intraperitoneal (IP) regimen limitation of post-operative adhesions. Hyperthermia itself has cytotoxic effects and can potentiate antineoplastic effects of chemotherapy in part by increasing the depth of tumor penetration by up to 3 mm. For the treatment of ovarian cancer, HIPEC has been evaluated in the recurrent setting with secondary cytoreduction. Recent studies, including a prospective trial, have evaluated its role in primary management of ovarian cancer. This review summarizes previous and ongoing studies regarding the use of HIPEC in the management of ovarian cancer.

## 1. Introduction

Epithelial ovarian cancer (EOC) is the deadliest gynecologic malignancy [1]. The majority of women are diagnosed at advanced stage with widely metastatic peritoneal disease. Standard of care involves a combination of surgery and chemotherapy. The ability to surgically resect tumors with optimal cytoreduction surgery (CRS), ideally to no gross residual disease (R0), is an important positive prognostic factor [2]. Despite the improvements seen in median survival time with the current standard of radical tumor CRS and IV carboplatin and paclitaxel, long term survival rates for patients with advanced epithelial ovarian carcinoma remain disappointing and efforts continue to develop more effective primary therapy. 

For most patients with EOC, the majority of disease burden is in the peritoneal cavity and can be quantified by the peritoneal cancer index (PCI) [3]. The PCI is a measure of the extent of disease burden in the peritoneal cavity. Due to this location, normothermic IP chemotherapy has been studied in prospective clinical trials in the post-operative treatment of primary EOC, and NCCN has noted the combined IV/IP regimen as preferred regimen for optimally cytoreduced Stage III EOC. In the setting of recurrence, treatment guidelines are determined by the time to recurrence and location of metastatic disease. HIPEC during CRS for EOC has been gaining more attention in the treatment of metastatic peritoneal disease. Specifically, HIPEC has more frequently been utilized in the recurrent setting with secondary CRS, but recent studies have evaluated its role in primary management of ovarian cancer. The aim of this article is to review previous and ongoing studies regarding the use of HIPEC in context of the overall use of IP chemotherapy for the treatment of EOC. 

## 2. Normothermic Intraperitoneal Chemotherapy

In normothermic IP chemotherapy, cisplatin and paclitaxel are injected into the patient’s peritoneal cavity through an intraabdominal port. IP chemotherapy is administered in the post-operative period over a course of up to six cycles. Three large prospective randomized studies support the use of IP chemotherapy in the primary treatment of EOC. In the Gynecologic Oncology Group (GOG) 104 study, patients were randomized to two arms: the control arm of cisplatin and cyclophosphamide IV and the experimental arm of cisplatin IP and cyclophosphamide IV. While there was a statistically significant overall survival (OS) benefit to the IP regimen of 49 months in comparison to 41 months for the IV regimen, consensus was the benefits of IP chemo are not greater than the benefits of new agent paclitaxel [4]. In GOG 114, patients in the control arm received six cycles of cisplatin and paclitaxel IV with an OS of 52.5 months and the experimental arm received two cycles of carboplatin IV, followed by six cycles of cisplatin and paclitaxel IP with an OS of 63.2 months. Progression free survival (PFS) and OS were statistically significant, but were partially attributed to the addition of two extra cycles of chemotherapy in the IP arm [5].

GOG 172 influenced practice patterns in the United States. The IV/IP regimen of IP cisplatin and paclitaxel, plus IV paclitaxel demonstrated the longest median OS compared to IV carboplatin and paclitaxel in patients with optimally cytoreduced stage III ovarian cancer. The median PFS for the IV alone and IV/IP regimens was 18.3 and 23.8 months, respectively. The median OS for the IV and the IP regimens was 49.7 and 66.9 months, respectively. Due to chemotherapy-associated toxicities, only 42% of women on the IP regimen actually received six cycles of therapy, and 49% received three or fewer IP cycles [6]. Because the OS benefit outweighed the toxicity of the regimen, the NCI Clinical Announcement recognized the superiority of IP chemotherapy in the optimal disease setting [7].

In a follow up analysis of the mature data of GOG 114 and GOG 172 combined, an OS benefit remains significant for IP regimens after 10 years of follow up. This benefit in OS was most pronounced in patients who underwent optimal CRS to R0 treated with the IP regimen. Specifically, in GOG 172, the OS was 127 months in this subset of patients [8]. There was also a correlation noted between survival and the number of IP cycles completed in a separate follow up analysis [9]. 

Despite the favorable OS for IP chemotherapy, the IP cisplatin-based chemotherapy regimen has not been universally accepted as a standard treatment for EOC secondary to regimen toxicity and IP catheter access device problems. A more recent large prospective trial, GOG 252, compared weekly IV chemotherapy regimens to varying dose reduced IP regimens. All arms of the trial had bevacizumab added during treatment and as maintenance. No significant differences in PFS were observed between the three arms. In comparison to GOG 172, more patients were able to complete the IP regimens, but all arms had excessive toxicity. One concern in interpreting the data from GOG 252 is the addition of bevacizumab to all arms could have influenced the results and analysis [10]. With the inability to replicate the results from GOG 172 and the limitation to access IP chemotherapy outside of the tertiary setting, there has been increased interest in HIPEC as a treatment alternative in the primary and recurrent ovarian cancer setting.

## 3. HIPEC

In HIPEC, heated intraabdominal chemotherapy is administered at the time of CRS. HIPEC has several potential benefits. High-dose chemotherapy can be used because the plasma-peritoneal barrier results in little absorption into the blood stream [11,12]. In addition, there is higher peritoneal penetration in comparison to IV regimen, and HIPEC does not have the limitation of traditional IP regimen of post-operative adhesions [13,14]. Hyperthermia itself has cytotoxic effects and can increase the depth of tumor penetration by the chemotherapeutic agent up to 3 mm and moreover can potentiate its antineoplastic effects [15,16,17,18].

A major historic limitation to HIPEC is the previously reported morbidity and mortality and thus its use was often discouraged [19]. To proceed with HIPEC, CRS to R0, CC0 (non-visible disease remaining) or CC1 (less than 2.5 mm visible disease remaining) is required and involves radical and complex surgeries that are associated with higher complication rates. Currently, particularly in high-volume centers with HIPEC specialists, morbidity and mortality has drastically improved [20,21]. One large retrospective review of 694 patients, treated between 2005 and 2011, utilizing the American College of Surgeons National Surgical Quality Improvement Program (ACS NSQUIP) database, demonstrated a complication rate of 33% and 30-day mortality of 2.3%, both rates consistent with outcomes for other major complex abdominal operations [21].

In EOC, HIPEC has been evaluated in the primary and recurrent setting. The majority of published data regarding this treatment modality is retrospective, but recently some prospective data has been published. Here we will review study outcomes with HIPEC in the management of primary and recurrent ovarian cancer as well as review ongoing trials. 

## 4. HIPEC in the Primary Treatment of Ovarian Cancer 

The largest prospective randomized clinical trial demonstrated a survival advantage for patients who received HIPEC, compared to standard IV chemotherapy, for the treatment of primary EOC. (Table 1) All patients received neoadjuvant chemotherapy after determining they were not eligible for primary CRS and had to have at least stable disease after receiving up-front IV chemotherapy. The control arm received standard IV chemotherapy before and after CRS (PFS = 10.7 months, OS = 33.9 months). The experimental arm received the same standard IV chemotherapy but also received HIPEC with cisplatin during CRS (PFS = 14.2 months (*p* = 0.01), OS = 45.7 months (*p* = 0.02)). Over 90% of patients completed full six cycles of IV chemotherapy in both arms [22]. While the PFS and OS in this trial are shorter than the previous mentioned normothermic IP trials, it should be noted that this is a different patient population. The PFS and OS survival in the control arm of this trial are similar to established data in patients receiving NACT and interval CRS [23]. Similarly, a large retrospective study from Italy showed improved outcomes in patients who underwent HIPEC after having a complete or partial response to neoadjuvant IV chemotherapy in comparison to HIPEC at primary CRS. [24,25] In addition to standardizing the HIPEC procedure, the time of administration of HIPEC is another important factor. 

A retrospective cohort study from France looked at 92 patients receiving HIPEC for primary EOC treatment. The majority (60.8%) received consolidation HIPEC treatment after receiving 6–9 cycles of IV carboplatin and paclitaxel. The rest received HIPEC at primary CRS (13%) and at interval CRS (26.1%.) The majority of patients received cisplatin HIPEC (80.4%,) but 35.9% did receive a second agent with HIPEC, either doxorubicin (19.6%) or mitomycin (18.5%). Significant to survival were timing of HIPEC, peritoneal cancer index (PCI), and R0 CRS. Longest median OS was seen in the primary CRS group at 52.7 months, followed by interval CRS at 36.5 months and then consolidation HIPEC at 33.4 months (*p* = 0.03.) Of all primary HIPEC patients, those able to be optimally cytoreduced to less than 2.5 millimeters (mm) had a median survival of 41.5 months compared to 21.2 months in those with residual disease greater than 2.5 mm (*p* < 0.01) [26]. Again, this is a different patient population than was evaluated in previous normothermic IP trials; therefore we cannot make direct comparisons. 

A trial from Spain prospectively evaluated a smaller series of primary, first recurrence and second recurrence EOC patients. Fifteen patients received HIPEC in the primary setting, and all received neoadjuvant chemotherapy. All patients received a combination of cisplatin and doxorubicin chemotherapy during HIPEC. The majority (73%) of patients were optimally cytoreduced to no gross residual cancer, and the median OS was remarkably 77.8 months in this patient population. This survival is similar to previously published normothermic IP chemotherapy data, but, again, we cannot compare such a small series of patients with different parameters [27]. 

Another larger trial from Spain was a case control series evaluating HIPEC in both the primary and interval CRS setting. Twenty three patients underwent primary cytoreduction with HIPEC and 29 patients underwent neoadjuvant chemotherapy and then interval CRS with HIPEC. All patients had CC0 CRS to no visible residual disease. Interestingly, the PCI was significantly higher in the HIPEC arm meaning that these patients had a larger tumor burden at the beginning of surgery. Also, a higher rate of bowel anastomosis and peritoneal stripping was observed in the HIPEC arm, but these cases were performed after data was published showing that aggressive CRS is associated with improved survival. In contrast, most of the control arm cases were performed before this time period. While the ovarian histology was not categorized, they did identify tumor grade. Up to 30% of tumors in the HIPEC arm were low grade which is a higher than typical ratio. No information was provided of how many cycles of IV chemotherapy was completed. While unable to complete analysis of OS, the disease free survival (DFS) was followed at 1, 2 and 3 years. In the control arm, respectively, the DFS was 66%, 33%, 18%; and in the HIPEC arm, the DFS was 81%, 67%, 63% (*p* < 0.01). It was noted that the survival benefit of HIPEC was not significant in undifferentiated tumors [28].

A retrospective review from South Korea evaluated the role of HIPEC as consolidation treatment at the end of primary IV chemotherapy. All patients underwent primary CRS (included both CC0 and suboptimal patients in analysis) then received adjuvant IV chemotherapy. Patients then underwent a planned secondary CRS. There were 29 patients in the control arm and 67 in the HIPEC arm. HIPEC patients received either single agent carboplatin or paclitaxel at time of CRS. Early stage EOC did not show a survival advantage with HIPEC treatment. However, for stage III control and HIPEC patients, PFS at 3 years was, respectively, 16.7 % and 56.3% (*p* < 0.01) and OS 32.8% and 66.1% (*p* < 0.01.) There was no survival difference between the carboplatin HIPEC and paclitaxel HIPEC subgroups. A higher hematologic toxicity was seen in the carboplatin HIPEC arm, however [29].

## 5. HIPEC in the Treatment of Recurrent Ovarian Cancer 

Substantially more studies have been published regarding the use of HIPEC in the management of recurrent ovarian cancer. Although, a significant amount are retrospective, evaluating a small series of patients or inconsistent with patient parameters and HIPEC dosing. Platinum agents are one of the most commonly used during HIPEC for ovarian cancer, but the dose varies in trials. A phase I trial was published regarding the maximum tolerated dose of (MTD) of cisplatin for HIPEC at time of first recurrence (Table 2). The MTD established was 100 mg/m^2^ with 25% of patients experiencing Gr 3–4 toxicity. Notably no severe hematologic toxicity at this dose, and over 90% of patients completed all 6 cycles of adjuvant IV chemotherapy. The median PFS of 13.6 months was comparable to previously published PFS in recurrent ovarian patients treated with IV chemotherapy alone. Peritoneal platinum concentration was significantly elevated in comparison to plasma levels, and platinum DNA adducts were found in tumor biopsies after HIPEC confirming cytotoxic activity immediately after a single dose of cisplatin. A Phase II trial is currently open to further evaluate the efficacy of this dose and regimen [30].

The retrospective cohort study from France also looked at the role of HIPEC in recurrent ovarian cancer. The paper included 247 chemo-sensitive (defined as a recurrence interval of greater than six months after completing IV chemotherapy) and 223 chemo-resistant (defined as a recurrence interval of less than six months) EOC patients. Similarly, the majority of patients received cisplatin HIPEC (75.3%,) but 36.4% did receive a second agent with HIPEC, either doxorubicin (28.1%) or mitomycin (9.1%). Significant to survival were lower PCI and CC0 CRS. Longest median OS was in patients with PCI score of 0–8, 59.3 months, followed by patients achieving CC0 CRS, 51.5 months. Interestingly, there was not a significant difference in survival between the chemo-sensitive (42.2 months) and chemo-resistant (48.0 months) subgroups. This could signify the benefit of hyperthermia in chemo-resistant tumors [26]. Other studies, however, have shown no benefit to HIPEC in chemo-resistant patients, and this needs to be further evaluated [25]. 

The previous trial from Spain prospectively evaluated a smaller series of primary, as well as 19 first recurrence and eight second recurrence EOC patients. All patients received a combination of cisplatin and doxorubicin chemotherapy during HIPEC. The majority (74%, 75% respectively) of patients were optimally CRS to CC0. The median OS was 62.8 months in the first recurrence group and 35.7 months in the second recurrence group. There was no difference in survival between patients reduced to no gross residual disease (CC0) and those with less than 2.5 mm of disease (CC1) [27]. The survival in this study is similar to previously published data of patients being treated with secondary surgery for recurrent ovarian cancer [31,32].

In a second trial from Spain, a case control review was performed on chemo-sensitive disease at first recurrence. Chemo-sensitive defined as recurrence greater than 12 months from completion of treatment. Twenty two patients underwent CRS solely and 39 patients underwent CRS with HIPEC. All patients included underwent CC0 CRS to no residual disease. Median PFS was 22 months in the CRS alone group and 21 months in the CRS with HIPEC group. While both groups were optimally cytoreduced, the HIPEC had a significantly higher PCI score. This could indicate a more aggressive group of tumors and explain the similar PFS even with the addition of HIPEC. Also, paclitaxel rather than a platinum agent was used in the trial, and, due to the cell cycle dependent mechanism of action, it was theorized that it may not be the most effective agent for use during HIPEC. Reassuringly, both groups had similar post-operative toxicity [33].

A similar patient population was studied in Italy. A case control study with 37 patient controls receiving either CRS and IV chemotherapy (13 patients) or IV chemotherapy alone (24 patients) versus 30 patients undergoing CRS and HIPEC. All patients were experiencing a first recurrence, and the initial PFS was similar in both the control and case arms. The only significant difference between the arms was pattern of recurrence. The control arm had significantly more patients with single nodule or localized recurrence. All control patients achieved CC0 CRS, and 96.7% of HIPEC patients achieved CC0 CRS. PFS was 15 months in the control arm and 26 months in the HIPEC arm. Interestingly, over half of the HIPEC patients had a longer secondary PFS after HIPEC than the primary PFS after initial treatment. The HIPEC patients had significantly longer OS, secondary PFS, and deaths than the control group [34].

A prospective trial from Greece evaluated the role of HIPEC at first recurrence. Sixty patients were randomized to each arm; CRS followed by IV chemotherapy versus CRS with HIPEC followed by IV chemotherapy. The trial included both chemo-sensitive and chemo-resistant patients. The HIPEC chemo-sensitive patients were treated with cisplatin and paclitaxel during CRS and the chemo-resistant were treated with doxorubicin and paclitaxel or mitomycin. Mean OS was 26.7 months in the HIPEC group versus 13.4 months in the control group (*p* < 0.01.) The OS was similar in both the HIPEC chemo-sensitive (26.8 months) and chemo-resistant (26.6 months) subgroups. In comparison, the OS was significantly different in the control arm chemo-sensitive (15.2 months) and chemo-resistant (10.2 months) subgroups (*p* < 0.01.) Both arms achieved similar rates of CC0 CRS. However, the overall survival in the HIPEC CC0 group was significantly higher (30.9 months) than the control CC0 group (16.9 months) [35].

## 6. Discussion

Ovarian cancer is the deadliest gynecologic malignancy in the United States. Normothermic IP chemotherapy for primary EOC has a known benefit in the optimal CRS setting. Unfortunately, widespread use has not occurred due to concern for toxicity and patient access to tertiary care centers. Due to these concerns, there is interest in HIPEC therapy for the management of primary and recurrent EOC. 

The largest HIPEC study published to date was in the setting of primary EOC. A survival benefit in patients undergoing interval CRS was found with the addition of HIPEC, and there was no difference in toxicity between the control and HIPEC arms [22]. A critique of the study is that it did not have an IP chemotherapy arm for comparison. The role of normothermic IP chemotherapy is unclear in the interval CRS patient population. A phase II randomized trial, OV21/PETROC, was completed and the IP regimen was found to be well tolerated with reasonable toxicity and no reduction in QOL. There was a noted decrease in progression of disease at nine months in the IP group, however, as the study was underpowered, there was no difference found in PFS and OS between the IV and IP arms [36]. 

More studies have been published in the recurrent setting, however, most are small and retrospective. A primary critique of HIPEC therapy in EOC is that there is not a standardized regimen. Platinum agents, specifically cisplatin, are frequently used but at varying doses. The phase I trial published defining cisplatin 100 mg/m^2^ as the maximum tolerated dose (MTD) will be important to consider when moving forward with designing HIPEC trials in EOC. This was the same dose utilized in the above mentioned primary EOC prospective trial. 

Along with varying doses in the recurrent setting, there were varying responses to HIPEC therapy. Prolonged disease free intervals have been shown in both the first and second recurrence settings. Interestingly, some trials have shown similar response in both chemo-sensitive and chemo-resistant recurrences [26]. In one study, the HIPEC arm of patients had a significantly higher PCI at time of CRS yet similar survival to the control arm [33]. A higher PCI is concerning for a more aggressive tumor biology, and could mean that the HIPEC played a role in the similar survival. Overall, there has been a positive significant survival response to HIPEC in the recurrent setting, but almost all published data is from small, retrospective studies.

A significant concern of HIPEC is the toxicity associated with the regimen. Prospective data published shows HIPEC to have similar toxicity to CRS followed by IV therapy [22,33]. Again, these are a limited number of studies, and further evaluation of morbidity and mortality needs to be performed. Another concern of HIPEC therapy is the increased cost associated with frequent ICU admissions and length of hospital stay. The inpatient IP regimen was found not cost effective in the short term in comparison to the traditional IV regimen, but when long term survival analysis was considered it became more cost effective due to the improved survival [37]. There has been no cost analysis performed for HIPEC in EOC. The addition of targeted or immunotherapies to IV regimens is another popular treatment option being considered. The addition of bevacizumab has been found not cost effective when considering all advanced stage EOC receiving IV therapy [38]. However, the cost effectiveness of bevacizumab was improved when looking at a subgroup of patients [39]. This illustrates the significance of identifying appropriate patient populations for specific treatment modalities. It will be important in future trials to perform comparative cost analysis, especially if survival outcomes are similar. 

## 7. Conclusions

In conclusion, there is now high quality prospective data suggesting a survival benefit to HIPEC therapy for patients undergoing primary treatment of EOC after receipt of neoadjuvant chemotherapy and optimal cytoreduction. Poorer quality data exists supporting its use in other clinical contexts such as recurrent disease. This treatment has not been studied in multiple clinical contexts, the regimen and toxicity management has not been standardized and HIPEC has not yet been compared to other standard treatments such as normothermic IP chemotherapy. Therefore, the treatment of EOC with HIPEC outside of clinical trial would not be recommended. Further trials are undergoing (Table 3) and are needed to assess the appropriate patient population and mechanisms of action for HIPEC therapy. 

## Figures and Tables

**Table 1:** HIPEC primary trials.

Author	Study type	N ^1^	Chemotherapy	PFS	OS
Van Driel, et al. [23]	Prospective	245	Cisplatin	14.2 months	45.7 months
Bakrin, et al. [26]	Retrospective cohort	92	Cisplatin (80%) ^2^	n/a	CC0: 41.5 months
Gonzalez Bayon, et al. [27]	Prospective	15	Cisplatin and Doxorubicin	n/a	77.8 months
Cascales-Campos, et al. [28]	Retrospective Series	52	Paclitaxel	1 year: 81% 3 years: 63%	n/a
Bae, et al. [29]	Retrospective Case Control	67	Carboplatin or Paclitaxel	3 years: 56.3%	3 years: 66.1%

^1^: Number of HIPEC patients in trial. ^2^: Chemotherapy included in analysis: included cisplatin, doxorubicin, oxaliplatin, mitomycin, cisplatin and mitomycin, and cisplatin and doxorubicin

**Table 2:** HIPEC recurrent trials in ovarian cancer.

Author	Study type	N ^1^	Chemotherapy	PFS	OS
Zivanovic et al. [30]	Phase I prospective	12	Cisplatin	13.6 months	n/a
Bakrin et al. [26]	Retrospective Cohort	470	Cisplatin (76%) ^2^	n/a	CC0: 51.5 months
Gonzalez Bayon et al. [27]	Prospective	27	Cisplatin and Doxorubicin	n/a	1st recurrence: 62.8 months 2nd recurrence: 35.7 months
Cascales-Campos et al. [28]	Case control	39	Paclitaxel	21 months	n/a
Fagotti et al. [34]	Case Control	30	Oxaliplatin	26 months	5 years: 42.7%
Spiliotis et al. [35]	Prospective	60	Multiagent ^3^	n/a	26.7 months

^1^: Number of HIPEC patients in trial. ^2^: Chemotherapy included in analysis: included cisplatin, doxorubicin, oxaliplatin, mitomycin, cisplatin and mitomycin, and cisplatin and doxorubicin. ^3^: Chemo-sensitive—Cisplatin and paclitaxel; Chemo-resistant—Doxorubicin with paclitaxel or mitomycin

**Table 3:** Ongoing randomized HIPEC trials in ovarian cancer.

Country	PI	Phase	Time Point	Sample Size	Chemotherapy	Clinicaltrials.gov Identifier
South Korea	Chang	N/A	Primary	204	Paclitaxel	NCT03448354
United States	Momeni	1	Recurrent	20	Carboplatin	NCT02672098
Italy	Not provided	N/A	Recurrent	158	Cisplatin	NCT01538785
Spain	Villarejo Campos	3	Primary or recurrent	94	Paclitaxel	NCT02681432
China	Cui	3	Primary or recurrent	214	Paclitaxeland cisplatin	NCT03373058
United States	Jewell	2	Primary	20	Cisplatin	NCT03321188
Italy, Germany	Ansaloni	3	Primary	94	Cisplatin and paclitaxel	NCT01628380
Mexico	Salcedo-Hernandez	2	Primary	100	Cisplatin and doxorubicin	NCT03275194
Spain	Villarejo Campos	3	Primary or recurrent	32	Cisplatin	NCT02328716
Belgium, France, Spain	Classe	3	Recurrent	444	Cisplatin	NCT01376752
France	not provided	3	Recurrent	220	Cisplatin	NCT03220932
United States	Zivanovic	2	Recurrent	98	Carboplatin	NCT01767675
India	Solanki	N/A	Primary or recurrent	150	Not provided	NCT02754115
United States	Sardi	2	Primary	48	Carboplatin	NCT02124421
United States	Kelly	2	Primary or recurrent	40	Carboplatin	NCT03188432
United States	Dellinger	1	Primary or recurrent	5	Cisplatin	NCT01970722
Norway	Flatmark	Observational	Primary or recurrent	200	Not provided	NCT02073500
Belgium	Ceelen	2	Primary or recurrent	48	Cisplatin	NCT02567253
United States	Lilja	2	Recurrent	200	Cisplatin	NCT02349958
France	Bereder	N/A	Primary or recurrent	44	Not provided	NCT02803515
